# Relative solidarity: Conceptualising communal participation in genomic research among potential research participants in a developing Sub-Saharan African setting

**DOI:** 10.1371/journal.pone.0195171

**Published:** 2018-04-05

**Authors:** Olubunmi Ogunrin, Kerry Woolfall, Mark Gabbay, Lucy Frith

**Affiliations:** 1 Department of Medicine, University of Benin, PMB, Benin City, Nigeria; 2 Biomedical Ethics Research Group, Department of Health Services Research, Institute of Psychology, Health and Society, University of Liverpool, Liverpool, United Kingdom; 3 Department of Psychological Sciences, Institute of Psychology, Health and Society, University of Liverpool, Liverpool, United Kingdom; University of Cape Town, SOUTH AFRICA

## Abstract

**Objective:**

As genomic research gathers momentum in sub-Saharan Africa, it has become increasingly important to understand the reasons why individuals wish to participate in this kind of medical research. Against the background of communitarianism conceived as typical of African communities, it is often suggested that individuals consent to participate on the grounds of solidarity and to further the common good. In this paper, we seek to explore this contention by presenting data from focus groups with potential research participants about what would influence their decisions to participate in genomic research.

**Methods and results:**

These focus groups were conducted as part of a larger qualitative study with a purposively selected group of participants from a community situated in south west Nigeria. We conducted fifteen focus group sessions comprising 50 participants organized by age and sex, namely: 1) adult (>30 years) males, 2) adult females, 3) youth (18–30 years) males, and 4) youth females. A mixed age-group was conducted to probe different views between the age groups. There was discordance and clear division between the adults and youths regarding the decision to participate in genomic research based on commitment to communal values. Adults based their decision to participate on altruism and furthering the common good while youths based their decisions on personal benefits and preferences and also took into account the views and welfare of family members and neighbours.

**Conclusions:**

This discordance suggests a ‘generational shift’ and we advance a model of ‘relative solidarity’ among the youths, which is different from the communal solidarity model typical of African communitarianism. Our findings suggest the need for a closer look at strategies for implementation of community engagement and informed consent in genomic research in this region, and we recommend further studies to explore this emerging trend.

## Introduction

Genomic research activity has grown in sub-Saharan Africa over the last decade and a half. The H3Africa Consortium was established to catalyze research about human diversity and genomics of relevance and benefit to African populations and societies [1]. One of the major challenges to successful implementation of genomic research in sub-Saharan Africa is developing culturally appropriate means of engaging with potential research participants and communities prior to, during, and after collection of biological samples and data for storage (biobanking). In a report published in Human Genomics, Ramsay et al (2014) observed that the primary ethical challenges to genomic research in Africa pertain to sensitivities and questions raised by communities based on their prior experiences as well as regional cultural beliefs and practices [2]. In the same vein, Tindana et al showed that genomic research stakeholders in Ghana and Kenya were concerned about culturally appropriate consent for sample export and reuse, and there was a need to understand cultural sensitivities around the use of blood samples [3]. These reports drew attention to the influence of community-related ethical issues on the willingness of individuals in African communities to participate in genomic research. Therefore, to address such ethical challenges and potential conflicts, it is important to understand the preferences and attitudes of the potential research participants to genomic research, what informs their decisions to consent or refuse to participate in genomic research by donating biological materials for biobanking and potential implications for ethically robust governance of genomic research in sub-Saharan Africa.

### Background

Previous research has highlighted the importance of the cultural dimensions that influence people’s decisions about whether to consent to participate in medical research. In the United States, for example, a community-based participatory research project among a black African immigrant community, explored perceptions about genomics, barriers and facilitators to participation, and ethical ways of engaging communities. The study reported that prominent in the participants’ views was the legacy of colonial mistreatment and exploitation by Western researchers in their home countries in sub-Saharan Africa. Hence the authors recommended a community-based participatory research model for genomic research; by sharing power with communities in setting the research agenda, designing biobanks through effective community engagement and assuring the rights and privileges of the community members [4]. In contrast, a study conducted in a different context to assess and identify the factors associated with the public’s willingness to participate in genomic research among a Swedish population, found that most people had a positive attitude towards biobanking research, and those more likely to donate body samples were middle-aged men who were mainly driven by altruism [5]. These studies illustrate a diversity of opinions among people from different communities, and the cultural location of people’s experiences clearly affect how research participation is framed and the willingness to participate. Such diversity underscores the importance of investigating the opinions and views of individuals in specific contexts to understand what informs their decisions to participate in genomic research.

There is a small but growing literature on informed consent to genomic studies in sub-Saharan Africa that has important implications for understanding the consent process [6–10], and our study contributes to this. A review of publications on genomics research participation among communities in sub-Saharan Africa, between 2002 and 2016, found that most were from South Africa [10–14], two from Nigeria [15,16] and one from Ghana and Kenya [3].

This overview of the literature from sub-Saharan Africa showed different reasons why potential research participants would want to participate in medical research. For example, a qualitative study of South African Tuberculosis research participants on sample storage and re-use highlighted trust as an important factor in their willingness to consent [12]. In another South African study of research participants, some had donated blood samples both for altruistic reasons and personal benefits, and some were ignorant of the reasons why the samples were being collected, hence signifying a lack of fully informed consent [13].

In the Nigerian context, one study reported positive attitudes towards genomic testing irrespective of age, literacy level or sex. However, participants claimed that aspects of biobanking that contradicted their religious beliefs and cultural practices affected their attitudes to genomic research [15]. Another study reported that altruism motivated the study participants [16]. The disparities in outcome of these two studies from the same country underscore the relevance of exploring in greater depth what factors influence potential research participants’ decision to participate in genomic research.

#### African ethics

It has been argued that decision-making processes in the African community are underpinned by ‘African ethics’, an ethics characterized by communal or social autonomy as opposed to individual autonomy [17]. This concept of African ethics advocated by African bioethicists emphasize that, contrary to liberal individualism which has governed, to a large extent, health decision-making and consent for research participation in western countries, brotherhood, togetherness, solidarity and overall good of the community influenced individual decision-making among Africans [17,18]. In Africa, communitarian ethics is rooted in and flows from the communities’ traditional values and these values are important for moral decision making.

Communitarianism affirms that individuals are socially embedded, implying that personhood is woven into the community, and furthering the communal good takes precedence over individual gains. Hence, the common maxim among the Yorubas of south west Nigeria, ‘eniyan ni aso mi’ means ‘people are my clothes (coverings)’ portrays communality. This emphasizes that for a person to flourish, his success can only come through the support of others. It is an ancient African concept upon which certain traditional African values such as communalism and interdependency are built [19–22]. This concept has become defined as African communitarianism.

It is therefore the cultural norm in African societies for individuals to take decisions not based on their personal preferences but on due consideration of family members, community norms, and cultural beliefs, and for traditional community leaders (the monarch, chiefs and community elders or opinion leaders) to take decision on behalf of the community. In the light of this, bioethicists and researchers have suggested that the application of an ethics based on traditional autonomy-centered, liberal individualism to biobank governance is problematic in a sub-Saharan African setting. The principle of solidarity has been advanced as a plausible alternative that could work well in a sub-Saharan African context [23,24]. For example, in considering the willingness to participate in biobanking research, the Nuffield Council on Bioethics Report, ‘Reflections on an Emerging Concept in Bioethics’ proposed solidarity as a model for biobanking research globally, and specifically in sub-Saharan Africa [24,25]. This model was described as putting a stronger emphasis on harm mitigation and reflected people’s willingness to accept personal costs in order to assist others. Thus, they argued that solidarity and benefit sharing is a useful model for human genomics research in all settings [25].

The principle of solidarity is central to communitarian ethics and it places a special premium on the importance of the community over the individual. It is, however, not clear what influences individuals in sub-Saharan Africa to consent, or refuse consent, to participate in genomic research vis-à-vis donation, storage and export of their bio-specimens is needed, especially when the few studies from South Africa, Ghana and Nigeria have not examined the impact of communitarian ethics on the attitudes and perspectives of potential research participants in these countries.

Theoretically, we can expect African potential research participants to participate in genomic research and agree to biobanking of their bio-specimens based on altruism and solidarity because of the overriding influence of communitarianism. We wanted to explore how solidarity and core values of communitarianism would be utilized, or not, and how these concepts were employed in this context. This paper focuses on the findings from the focus groups conducted with potential research participants that pertained to commitment to communal values and willingness to participate in genomic research.

## Methods

Ethics approval were obtained from Lagos University Teaching Hospital research ethics committee in Nigeria (Reference number ADM/DCST/HREC/1792) and ethics committee of the University of Liverpool in United Kingdom (Reference number IPHS-1415-LB-270). The data reported here are part of a larger qualitative study into informed consent and community engagement in genomic research that explored the views and opinions of potential research participants and biomedical researchers in a sub-Saharan African community on consenting to genomic research participation and what factors influence their opinions on the donation of biological samples. We conducted a qualitative study using four focus group categories with 15 focus group discussion (FGD) sessions to obtain data from purposively selected community members. Qualitative research is valuable for studying what is important to people based on their knowledge and experience and seeks answers to questions that stress how social experience is created and given meaning [26]. Therefore, we chose FGD to explore beliefs and perceptions of our study participants and illuminate their opinions on genomic research participation.

### Study area

This project was situated at a tertiary health institution in a semi-urban community in south west Nigeria. It is a community of mostly Yoruba-speaking people with either Christian, Islamic or Traditional religious affiliation. Employment is both white-collar (salaried) and indigenous traditional occupations like hunting, farming, and crafts. The community is situated along the trade routes between ports in Niger delta and the Yoruba mainland.

### Study participants

Participants were purposively selected from among community members attending the research facility when they registered at the facility’s medical records department. Study participants were not participating in genomic research. They were categorized, based on their ages, into adults and youths. Adult participants were those above the age of 30 years, while the youths were between 18 and 30 (inclusive) years of age. The categorization of adult and youth participants using this age bracket was based on the national youth policy. All study participants resided within the community.

### Recruitment

One of the authors approached the FGD participants personally and through a community contact. Those who were literate and could read were given an information sheet and opportunity to ask questions about the research. Those who could not read were personally given the details of the research verbally and also given the opportunity to ask questions. Consent was obtained in the form of a signature or a thumb print after giving adequate time for each potential participant to study the information. As far as possible, the recruitment was done at the first contact as it is more difficult to follow up and get the potential participant to consent subsequently.

### Study design

Study participants were segmented by age and sex into four categories, namely adult males, adult females, male and female youths. We chose to have separate groups for the males and females. This categorization of participants aimed to minimize paternalism which may affect women and young persons from freely expressing themselves in a mixed group as noted previously by Fagbemiro *et al* [15]. The women were in a separate FGD category and were encouraged to talk freely, thus developing a deeper understanding of gender inequalities within the family setting and community, and how these affected their choices to participate in genomic research. The FGD was moderated by one of the authors (OO), a male and trained on conducting FGD, and a female research assistant (a Master’s graduate of Medical Sociology). There was also a female co-moderator, a staff member of the research institution who is from and familiar with the community, for the female FGD sessions.

We observed from the literature that youths were seldom included as participants in most qualitative studies on research participation conducted in sub-Saharan Africa and that this was, potentially, an important omission. A mixed group comprising adults and youths of both sexes, was conducted to probe discordant views among participants. This was done to ensure credibility and dependability through reflexivity [27,28].

A topic guide that contained questions on peoples’ willingness to participate in genomic research, what informs their decisions to participate, how they arrive at a decision, and what factors influence such decision was used for the FGD sessions [S1 File].

### Data collection

All the FGDs were held in a quiet, well-lit room in the medical outpatient department of Babcock University teaching hospital, Ilishan Remo, Nigeria. All participants had earlier agreed to a convenient time and arrangements to convey those who had challenges with transportation was made.

Before each session, participants were given a brief explanation of purpose of the FGD and asked to explain what they understood about genomic research and, then a definition of genomic research was given to them to establish a common starting point. Participants were encouraged to express their views, opinions and experiences on the topics discussed. All the sessions were audio-recorded and the participants agreed to this as part of the consent process, and notes were taken by one of the moderators or the assistant to supplement the recording. The notes were compared with the transcribed texts from audio-recorded FGDs. The data obtained were transcribed verbatim after each FGD for analysis.

### Data analysis

The methodological design was adapted from grounded theory [29] and the constant comparative method to develop themes was used for the data analysis [30,31]. Data were iteratively analyzed and as themes evolved during the analysis, these were further probed during the subsequent FGD sessions to achieve saturation and clarity of data. The coding of the data was aided by Atlas-ti qualitative software (Cincom Systems Inc., GmbH Berlin, 2016). The initial or open coding yielded themes which were subjected to selective coding to identify common and explanatory categories for common themes. Deviant cases were identified, discordant views were deliberated on. The quality of data analysis was ascertained by 1) expert reviews and several discussions of developing coding framework with supervisory team (KW, MG, and LF), 2) data or informant triangulation (that is comparing data obtained from different groups of participants on same issues of interests, for example we compared the responses from the various focus group discussants, that is the adult male and female participants, and the female and male youths), 3) code-recode analysis (this involved open-coding half of the data and then, re-coding same half and comparing the results of the two coding processes for similarities and differences), and 4) demonstrating reflexivity by categorizing the focus groups using gender and age, and a female co-moderator facilitating the female FGD sessions because of sensitivity to gendered and age effects on FGD outcome [32].

## Results

### Characteristics of participants

Fifty individuals, 24 females and 26 males, participated in 15 FGD sessions conducted with four to six participants at a time over a period of eight weeks. The adults participated in 6 FGD sessions, youths in 8 FGD sessions, and a mixed group of adults and youths in one session. This is a good-sized sample for qualitative inquiry [33]. Each FGD session lasted for a period of 45 to 75 with a median of 65 minutes. Participants were almost equally distributed by sex, with mean ages of 47.6 (range 35–64) and 24.6 (18–30) years for the adults and youths respectively. The background characteristics of the participants are shown in Table 1. Most were Yorubas with rest being either Igbo (9) or Hausa (1). The ten individuals who were non-Yorubas have lived most part of their lives in the community and become attuned to the community’s values and beliefs. Most of them were educated and employed, as either public employees or self-employed.

**Table 1:** Characteristics of participants.

	Focus Groups		
	Adults	Youths	Mixed group ^a^
Sex			
*Female*	10	12	3
*Male*	10	12	3
Age range (in years)	35–64	18–30	24–57
Marital status			
*Married*	20 ^b^	8 ^c^	4 ^d^
*Single*	0	16	2
Level of education			
*No education*	1	8	0
*Home tutoring*	1	2	0
*Primary* ^e^	8	5	0
*Secondary* ^f^	5	5	3
*Tertiary* ^g^	5	4	3
Employment status			
*Salaried*	5	3	3
*Self-employed*	10	14	2
*Pensioner*	3	0	0
*Unemployed*	2	7	1

^a^ Though the study participants were segmented by gender and sex, a mixed group of adults (above 30 years) and youths (between 18 and 30 years inclusive) without sex categorization was constituted as the last focus group to probe emerging themes for theoretical saturation.

^b^ all adults are married with children

^c^ married youths with children

^d^ the mixed group has three married adults and a married youth (female)

^e^ Primary level of education refers duration of schooling ≤ 6 years with no post-primary education.

^f^ Secondary level refers duration of schooling > 6 years but < 12 years with no post-secondary education.

^g^ Tertiary level refers duration of schooling ≥ 12 years (university or equivalent institutions).

Four major themes emerged from the data on the reasons and reasoning processes of prospective research participants when deciding to take part in genomic research.

### Benefits of research

Participants discussed how they considered the potential and immediate benefits of the genomic research to themselves before deciding whether or not to participate. Two youths said,

‘I will agree to testing either for research or therapy, and this time it is even beneficial and in the future, it will help me to prepare in case some disease is discovered’ (Youth male 7).

‘I want to be sure of what I will benefit from it, though I would still have a little fear, but I will agree if I know the benefits’ (Youth female 4).

Direct personal benefits identified included getting their genomic tests done at no cost, possible medical checks of their blood pressure and sugar and opportunity for individuals to know their genotypes, as expressed by two adult males and one adult female:

‘I think it will give me the opportunity especially in this environment where people do not really do medical checkup, medical check is something important for everyone to do, so if I am opportune to be called upon to partake in such opportunity more so it is free, I think I should oblige to it, and get back to my family to let them know.’ (Adult male 10).

‘Knowing (the result of test) helps you to prevent the disease. It is like getting to know that tomorrow the glass of your car will get scratched, so you ask yourself what will I do to prevent the glass from getting scratched, then I do something about it.’ (Adult male 4).

‘I would agree to participate, it will help us to know our genotype. Some people do not want to know their genotype, whether AS or SS because of fear of who will marry me, but it is good to know’ (Adult female 2).

Though there was consensus on considering personal benefits of research between the youths and adult participants, the adults talked more about the benefits to the larger community.

‘Benefits to the whole community is emphasized, not so much to the individual. Emphasis is on the community. What the researcher wants to extend to the people is for the community, since people will know what they will gain at the end of the exercise then we will agree. At the end of the day what benefit is extended to the people is for the community’ (Adult male 1)

‘First is to consider the community, that is the benefits to the community, so you must go to the ruler, and share what you want to do. The benefit of health to the community is important, even animals need health, without protecting its health it would just die’ (Adult male 2)

Some adults expressed the benefits to the community in the form of improvements in health delivery based on the outcome of the research and how the discovery of new drugs or vaccines could prevent diseases affecting the members of the community as illustrated by the views of this adult participant,

‘One of the things that encourages people to agree is the benefits to the community members like those with HIV or hepatitis will know if they have the disease, or even knowing our blood group. This type of research can help to make new drug or vaccine to treat these diseases and help people, so if people know about this they will come out, even those who cannot walk will find a way to come’ (Adult male 9)

Hence, adult participants appeared to be more committed to communal values and the future benefits of genomic research to the community than the youths (only four of the youths considered future benefits of genomic research to the community).

### Trust

Regardless of age, participants described how trust in the researcher and research institution were important factors when deciding whether to participate in genomic research. They stressed that since the researcher was a ‘stranger’ to the community, they needed to know and confirm the researcher’s identity, as well as the integrity of the research institution before deciding to participate.

‘It is important that they (researchers) are people we can trust, otherwise it would be difficult to convince the community to agree’ (Adult male 8).

‘Once I have confirmation (that research is genuine) then there is no problem in allowing them (researchers) to do what they want to do, maybe it will be used for testing and development of drugs so that they can establish the drug is genuine and will cure sickness so in that case once we have established (that they are genuine people), then we allow it, no harm since it is not for bad thing’ (Adult female 3).

‘It is difficult, one does not know the hospital the researcher is from, we live in a fearful world these days, one does not know a genuine researcher from a fake one, so they (researchers) should identify themselves’ (Adult female 3)

‘How do they identify themselves?’ (moderator)

‘They (researchers) need to interact with community leaders, then they (the community leaders) will call the community people’ (Adult female 2)

and the response of a male youth,

‘For medical research, if I trust the organization with my blood sample, I do not mind where they take it to so far they have my good intention at heart’ (Youth male 8).

They said they would have to trust the researcher not to use the samples collected for money-making rituals or voodoo practices to harm individuals, which according to some were common practice in the society. An example is the view of a female youth:

‘you are warned that anything pertaining to your blood just be very careful because at times doctors that even take it that we trust, we trust them but we don’t know what they are doing, because at the hospital they even take people’s blood, placenta do rituals, at times for you to release your blood, that will be very hard.’ (Female youth 3).

Adult participants, unlike most of the youths, also considered trust in the community leadership. They trusted community leaders to make decision on the behalf of their community.

‘You have to inform the community ruler and the chiefs, we have trusted leaders. Once you informed them you have, by so doing, informed the people’ (Adult male 4)

### Respect for community values and leadership

The majority of the adults, as distinct from the youths, based their willingness to participate in genomic research on communal values like respect for the decisions of the community leaders and family members, especially family heads. They associated their considerations of community leadership approval and opinions of family members with compliance with traditional practices and norms, meaning ‘to do it the way we have always done it in the community’. This was expressed in the words of two adult participants, one talked about substituting his individual choice for communal choice based on decisions taken by the community ruler who is perceived as the father of the community, inferring that individual autonomy becomes inappropriate in the face of communitarianism:

‘As long as I am staying within the king’s (oba’s) community, there is nothing I can do because he has given the order, so it is like a parent, parent (father) to the community, he is like a parent protecting his children, whatever he says should be respected, no matter how educated I am’ (Adult male 5).

‘The practice in this community is that the king approves on behalf of the whole community, this makes things easier’ (Adult female 8)

The adult females agreed that decisions to participate must be approved by their spouses who were the heads of the family. For example, an adult female said:

‘Father is the head of the home. The mother cannot just take decision without the father’ (Adult female 1).

The adult males talked about discussing their decisions with their wives before participating:

‘you can even start from your family, as an individual I have to consult my wife first, tell my wife that I want to do something like this get her personal opinion and add to my own considerations’ (Adult male 1).

The adult respondents emphasized approval of research by the community leaders and opined that this would protect the community from harm and exploitation. For example, they stressed the need to comply with community decisions, the decisions of the ruler and the elders who serve as ‘gatekeepers’ and therefore this would ensure community protection from fraudulent researchers. This underscores the recognition and influence of the existing societal authority structures in decisions over research participation.

‘The researchers should contact the community elders, who will confirm their identities. We must be sure if they are authentic. Once the elders informed us that they are genuine, then we will agree to donate our blood. It is what the elders decide’ (Adult female 4)

### Personal convictions

On this theme, there was a lack of consensus between the adult respondents and majority of the youths about whether to prioritize personal views when deciding to participate in genomic research. Most of the youths talked about participating in genomic research based principally on personal convictions unlike the adults.

‘I will consider how it affects me, does it affect me positively or negatively. So, if I am convinced then I will participate in the research’ (Youth male 13)

Also, some of the youths said they would consider the opinions of family members.

‘First is to consider how beneficial it is to me and then secondly the people around me, and thirdly I look at my background, my family background, whether my close family believe in it.’ (Youth male 2)

Some of them claimed that with their level of education they probably knew more about research than the leaders and, were more aware of the benefits and possible risks, so they would make their choices based on their personal convictions. For example, a male youth said:

‘In that case since I am more enlightened than the oba (the King) I will go ahead and do it, because I believe it will benefit me, and I will not just limit it to myself but I will also try to educate the people around me’ (Youth male 3).

This deviation from the communal norm prompted exploration in a bid to achieve theoretical saturation. This discordance was further probed using a focus group of mixed respondents (3 adults–one male and two females, and 3 youths–two males and one female). These focus groups were iteratively constituted to determine if the differences could be explained by demographic characteristics peculiar to either the youths or adults. We observed that youths who were artisans with no or primary level of education expressed similar responses as those with secondary or tertiary levels of education. We also compared the responses of youths who are unmarried with those who are married (with children) and adults (all adults who participated were married and had children), and this discordance remains. Summarily, there was no major division between youths who were married (and are parents) and those who were single, between those who were educated and those without formal education, and among their professional categories. We identified personal conviction based on being better informed through use of social media as a common denominator that influenced the youths’ views on participation in genomic research.

The session almost became confrontational as the youths insisted that, though they respected the position of the community ruler as being the ‘father of the community’, they were not bound to comply with his decision on genomic research participation. The majority of youth participants agreed that they would make a personal decision on genomic research participation. They attributed their stance to a) being better informed through the social media like the internet, b) personal preferences, and c) desire for personal benefits. Some youths talked about the influence of social media this way:

‘My own view, if I want to make a decision, first thing is I will consider how it affects me. Now we are in the jet age. People no longer go to ask for (other) peoples’ opinion because they will not be sincere, they will hide exactly what you need to know. So, I will go to assess internet sources, so I read about it on the internet, asking people will not be the best for me. On related matters, in the past, I took one week downloading videos and books, reading about it day and night to get my conviction I seem to be more confident when I have consulted all these sources. I decide on my own.’ (Youth male 13).

‘Now that most young people have mobile phones, we are better informed through connecting to internet, so problems of not being informed are solved. I can decide on my own, I do not need my family consent or someone else.’ (Youth female 9)

#### Differences in language

There was a notable difference in the use of language between the adult and youth participants. Adult participants used the words ‘community’ and ‘religion’ during their conversations reflecting emphasis on community values and benefits, unlike the youths who used the word ‘people’ rather than ‘community’ [see the word count in Table 2] and word clouds depicted by S1 Fig and S2 Fig.

**Table 2:** Word count for the adult and youth FGD participants.

Words	Frequency	
	Adult respondents	Youth respondents
**Community**	12	3
**Families**	9	15
**People**	15	24
**Religion**	13	3

## Discussion

The findings of our study showed that, although there was consensus about genomic research participation based on trust in the researcher and the research institution, and consideration of benefits of research, there was discordance between the adults and youths on their commitment to communal values as a motivating factor. This disparity reflects two ends of a spectrum. At one end, there is communitarianism, the position of the adults, and at the other end of the spectrum is a leaning towards autonomy or liberal individualism, the stance of the youths. The youths’ stance cannot be described as ‘pure’ liberal individualism because they expressed consideration for others, therefore they were not entirely expressing an individualistic stance. However, the youths sought liberty to make their own choices, and a commitment to equality in competence for decision-making. This did not, however, mean ignoring traditional community leadership and well-being of families and neighbours. On the other hand, the adult participants’ communitarian views emphasized the importance of common bonds between people. For example, the adult females would seek consent of their husbands before agreeing to participate, an observation previously reported by Marshall et al [6], while the adult males said they would discuss this with their spouses. This can be argued to reflect a philosophy that upholds the concept of societal welfare based on the foundational values of the collective good, common interests, solidarity, reciprocity and mutuality. This concept anchors the well-being of the individual and his or her identity within the social networks of the community. The communities build individuals just as much as individuals build communities [34,35]. The individual therefore cannot engage in deliberative actions without prior consideration of the communal interest.

To explain the disparities in the responses of the youths and the adults, we argue that there has been a ‘generational shift’. In a traditional community setting, the social networks are formed from traditional community leaders, families, religious groups, and professional groups (market women, hunters, farmers, etc.). Individuals within the community belong to and interact with one or more of these groups. Among the younger generations, there has been a significant growth in the use of the internet and social media tools, and these have had an impact on social networks within these communities. Social media forms part of the social connections that facilitate a different kind of social network by enhancing bonding, bridging and linking of the various actors within wider networks [36,37]. For example, social network sites enable interaction, and therefore reciprocity, with a larger network of social connections [38]. The youths also thought that their being more informed than the elders was due to their use of the internet, a practice more common in this age group than the older age group in sub-Saharan African communities.

A study by Ephraim (2013) showed that children and youths aged between 13 and 30 years constitute Africa’s heaviest users of social media [39]. This age group also constitutes Generation Y (people born between 1978 and 1989) which has been characterized as self-expressive, group-oriented, global and technology-dependent [40]. A rising tide of technologically sophisticated youth and expansion of education has been described by several authors, causing a generation gap and value-conflict with the adult world [40–42]. The influence of this generation gap has not been previously described in relation to genomic research participation. From our findings, this age-related shift, consequent upon exposure to ‘new’ technologies influenced the youths’ decisions and shaped the foundation of their willingness to participate in genomic research.

In communities, the multiple social networks that may be relevant for any individual, including families, workplace, and social, religious and political associations suggest that individuals are thinking about themselves in more complex ways. Social media tools, like the internet and mobile phones, provide a newly emerging mechanism for engaging a diverse group of individuals, form a diverse range of geographically situated communities, and provide a broader forum for discussion. These can facilitate the process of establishing collective positions and strategies where there is ready availability of social media tools among majority of the society, across different age groups. However, in communities like sub-Saharan Africa, where social media tools are not available across the various age groups the likelihood of significant knowledge gap exists. For those who use social media, their awareness of topical societal issues is enhanced thereby strengthening personal capacity to make individual decisions. Thus, these tools not only play an important role in building and sustaining wider international social networks by facilitating ongoing communication and exposure to other cultures particularly western cultures and values, but also influence decision-making on health-related issues like genomic research participation.

### Relative solidarity model

This discordance between the views of adults and the younger generation has significant implications for community engagement in Africa. It means that researchers must consider the views of different groups in communities as their perceptions and attitudes may differ based on their individual preferences and convictions. To presume that all people within a community will demonstrate similar ethico-social views may result in conflicts and disruption during the conduct of research. Therefore, to forestall ethical conflicts in the community engagement process for genomic research there needs to be a model that will embrace this phenomenon of ‘generational shift’ without disrupting the existing communitarian values and structure.

According to Callahan (2012), the first set of questions to be raised about any ethical problem should focus on its social meaning, implications and context even in those scenarios which only affect individuals [43]. Thus, to accommodate the position of the youths, we propose a model of ‘relative solidarity’. This model is a move towards a recognition of an individual’s capacity for agency and rationality without rescinding respect for communal values. This allows individuals to situate their personal opinions and preferences within the sphere of communal values thus embracing solidarity. This solidarity is relative, however, in that it considers the well-being of others without sacrificing individual choices and individual decision-making. The youths expressed concern that other community members should benefit from the genomic research. This is consistent with brotherhood, togetherness and communality.

We conceptualized the relative solidarity model within the ethical framework of responsive communitarianism, as opposed to authoritarian communitarianism. Authoritarian communitarianism in seeking the common good among societies, focuses on conformity and conventionalism, an authoritarian power structure, rigid stratification, and discriminatory practices against minorities and women, with enforcement of compliance to communal values through coercion and manipulation [44,45]. Consequently, authoritarian communitarianism rigidly enforces communal values on people without democratic dialogue and careful evaluation of what is good and bad about traditional practices, thereby causing severe restrictions on personal freedom, political and civil rights. This type of communitarianism broadly reflects the perceptions and views of the adults. Responsive communitarianism’s main thesis, on the other hand, is that people face a conflict of two major sources of normativity: that of the common good and that of autonomy and rights, neither of which in principle should take precedence over the other [34]. The model of relative solidarity we are proposing allows for expression of personal rights and preferences in furthering the common good, and is therefore not compatible with authoritarian communitarianism. However, it is compatible with responsive communitarianism that allows furthering of the common good without disregarding individuals’ preferences.

A delicate balance between liberty and social order, and individual rights and social responsibilities, is required to prevent ethical conflicts which may arise from the phenomenon of generational shift we have described. Our model of relative solidarity not only aligns with responsive communitarianism but, in addition, promotes respect for communal values without ignoring personal convictions in the furtherance of the common good of the community. Relative solidarity could foster a fruitful dialogue at community town hall meetings, ease tension that may arise from contrasting views among community members, and facilitate early resolution of conflicting ethical issues in genomic research participation. It can also offer a meeting point for communal solidarity that typifies responsive communitarianism and liberal individualism in African ethics when dealing with the ethical issues raised by genomics. See Table 3 for differences between communal and relative solidarity.

**Table 3:** Differences between communal and relative solidarity.

Goals	Communal solidarity	Relative solidarity
**Common good**	Pursuance is based on shared understanding of society and its goals (communal values)	Pursuance is based on personal convictions and communal values
**Obligation**	There is a strong sense of obligation to communal values	Limited obligation to those communal values that contradict personal convictions.
**Cost**	Willingness to bear costs on behalf of others towards achieving the common good	Willingness to bear costs on behalf of others towards achieving the common good, if these cohere with personal convictions

A relative solidarity model is a more useful conceptualization of our research participants’ view than relational autonomy, a concept that emphasizes autonomy as capacity of rational individuals to make un-coerced informed decisions without, at the same time, abrogating their obligations to other people [46].

Relational autonomy is simply the addition of communal values to a set of liberal values. Therefore, it is a modification of individual liberalism that sees individuals’ identities, interests, ends, and beliefs as fundamentally dynamic, continually constructed and reconstructed in dialogic processes with other people as well as with our traditions and with history [47]. Though it is grounded in the social nature of people’s lives, it remains a conception of autonomy. We believe this approach does not adequately reflect the existing communitarian ethic that is prevalent in African society.

Relative solidarity, on the other hand, is a modification of key communal values, therefore it is not outside the scope of communitarian ethics. A major difference between relative solidarity and relational autonomy is that relative solidarity allows for personal convictions over communal beliefs to further the common good, while relational autonomy allows individuals to bring their autonomous desires or choices to fruition, thus allowing autonomy to flourish. This implies that individual convictions within the framework of relative solidarity often align with achieving the common good, with benefits for everyone in the community. It is not individualistic, rather it questions traditionalism and beliefs that militate against community advancement and development. Therefore, in the context of genomic research, we propose that relative solidarity will motivate individuals to accept costs or risks of the research to further the common good based on personal convictions of the benefits of the research to the community.

### Limitations

A limitation of this study is that it only took place in one region of a sub-Saharan African country. Although the findings of this research may be transferable to other regions, this may be limited by, for example, the personal preferences and convictions of youths in other regions that cannot be presumed, and we cannot be certain about the availability or exposure of social media networks in other communities. Another limitation is the purposive sampling of participants that makes it difficult to generalize our findings. This was an exploratory study and further research is needed to more fully understand if and how the concept of relative solidarity is used in practice.

Despite these limitations, we recommend that genomic researchers involve various age groups in community engagement processes based on our generational shift concept. While seeking participation for genomic research, different approaches for recruitment may be advanced by researchers based on the understanding of the socio-cultural values and personal convictions of the potential research participants and communities. Sometimes these approaches may appear to conflict thus raising ethical concerns. For example, a researcher may choose to approach younger people individually and older people through a communal meeting during recruitment for a genomic research. The concept of relative solidarity can help justify these different recruitment strategies. Therefore, in developing policy guidelines for review and approval of genomic research protocols, the model of relative solidarity could be usefully employed by ethics committees and other stakeholders to resolve the ethical issues raised by genomic research participation. Further work needs to be done on how this could be achieved in practice.

## Conclusion

This study has shown a general willingness among potential research participants in a sub-Saharan African setting to participate in genomic research. There was discordance between the views of adults and youths on roles of communal values and personal convictions. The youths demonstrated a ‘generational shift’ from absolute solidarity of communitarianism, which embraces altruism, respect for traditional leadership, and mutual reciprocity, towards liberal individualism. We proposed a model of ‘relative solidarity’ within the ethical framework of responsive communitarianism as a way of interpreting this deviation from the norm.

Overall, understanding the factors influencing the willingness and attitude of individuals to participate in genomic research is a requirement for the success of this type of research. There has been no documentation of this phenomenon of generational shift in the literature from other African countries, to the best of our knowledge. We recommend further research is needed to explore this shift and how relative solidarity can be operationalized in approaches to consent in genomic research in other sub-Saharan African countries.

## Supporting information

S1 Fig Word cloud for adult FG participants. (DOCX)

S2 Fig Word cloud for youth FG participants. (DOCX)

S1 File FGD topic guide. (DOCX)
