# Correction: Relative solidarity: Conceptualising communal participation in genomic research among potential research participants in a developing Sub-Saharan African setting

**DOI:** 10.1371/journal.pone.0199514

**Published:** 2018-06-19

**Authors:** Olubunmi Ogunrin, Kerry Woolfall, Mark Gabbay, Lucy Frith

The following information is missing from the Funding and Acknowledgements sections: Professor Mark Gabbay and Dr Lucy Frith are part-funded by The National Institute for Health Research Collaboration for Leadership in Applied Health Research and Care North West Coast (NIHR CLAHRC NWC). The views expressed here are those of the author(s) and not necessarily those of the NHS, the NIHR, or the Department of Health and Social Care.
