# Supplementary figures and images for: Relative solidarity: Conceptualising communal participation in genomic research among potential research participants in a developing Sub-Saharan African setting
Source: PLoS One. 2018 Apr 5;13(4):e0195171. doi: 10.1371/journal.pone.0195171 (PMC5886479; doi:10.1371/journal.pone.0195171)

**S1 Fig. Word cloud for adult FG participants**


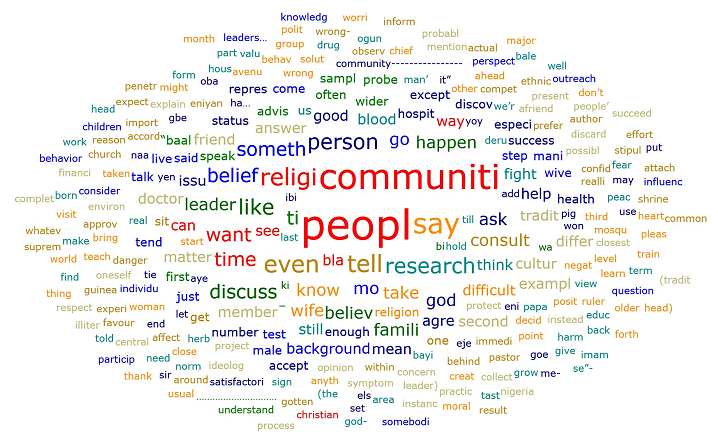

Supplement: S1 Fig — (DOCX) [file pone.0195171.s001.docx]

**S2 Fig. Word cloud for youth FG participants**


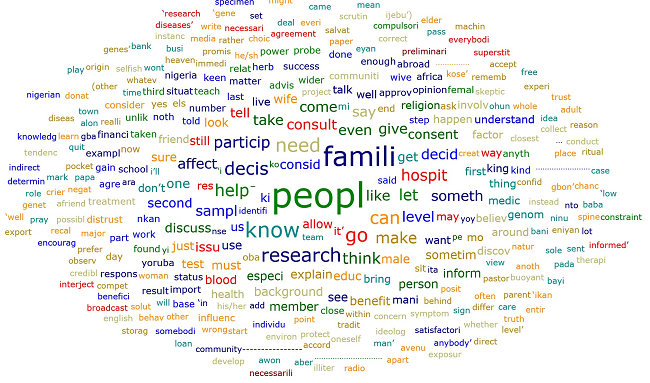

Supplement: S2 Fig — (DOCX) [file pone.0195171.s002.docx]
