# Supplementary material for: Relative solidarity: Conceptualising communal participation in genomic research among potential research participants in a developing Sub-Saharan African setting
Source: PLoS One. 2018 Apr 5;13(4):e0195171. doi: 10.1371/journal.pone.0195171 (PMC5886479; doi:10.1371/journal.pone.0195171)
Supplement: S1 File — (DOCX) [file pone.0195171.s003.docx]

**S1 File FGD topic guide**

**Demographic data of the study participants to include gender, level of income, ethnic orientation, religious affiliation, age and level of education**

This shall be obtained using a prepared format as presented below:

Age ………………. (years) Sex ………………. (Male/Female)

Domicile ……………………………………………………………………………

Level of education ……………………………. (No education, Home tutor, Primary, Secondary, Tertiary)

Ethnicity ………………………………… (Yoruba, Igbo, Hausa, others)

Religion …………………………………. (Islam, Christianity, African Traditional, others)

Level of Income …………………… (average per year in Naira, if applicable)

Role in the family ……………………………… FGD Category ……………………………

| S/N | Topic of interest | Question |
| --- | --- | --- |
| 1. | **Knowledge of genomic research** | Have you heard of ‘research’ before? Can you tell me what you understand by the word ‘research’? Have you heard of genomic research before? What does genomic research mean? How did you know about it? |
| 2. | **Importance of ethics in research in their communities** | Do you know what ethics mean? Do you think ethics is important in research? What is the significance of ethics in research? Can you explain why it is important? |
| 3. | **Process of decision making in the community** | If you have to participate in research how will you make a decision? Has there been any change in the decision making process? If yes, why and when? Is there any input to that decision from the community authority/family members/others? Are there other factors that influence your decision? |
| 4. | **Awareness of benefits and risks of research** | Do you think research is risky? What of genomic research? Can you tell me some of these risks? Can you give examples (if there is any)? Do you think there are benefits associated with research? What of genomic research? Can you give examples (if there is any)? How did you know about these benefits and risks? |
| 5. | **Impact of cultural and religious beliefs on research participation** | Does your religion affect participation of individuals in research? How does it affect participation? Does your culture encourage participation in research? Are there cultural practices or norms that affect your participation in research? What are these practices? How do they affect your participation? Do you think your gender/age group put you at a disadvantage as regards research participation? |
| 6. | **Knowledge of informed consent and who gives the consent** | What does ‘informed consent’ (agreeing to participate in research or any other project after you have received full information on what the project is about) mean to you? How much information will you need to give your consent for participation in genomic research? What do you think the process should entail? In genomic research, do you think informed consent is important? In genomic research, specimens may be used for other purposes apart those initially given. Do you think your consent for the initial purposes is sufficient to cover for the new purposes? If not, why? What will you want the researcher to do? |
| 7. | **Perception of export of donated specimens, ownership of such specimens and desirability for feedback following analysis of specimen** | Will you allow your specimen collected for research purposes be taken somewhere else in Nigeria for analysis? Will you export outside Nigeria for analysis? If not, why? Who do you think own specimens collected for research? Why do you think so? Will you want results of the analysis on your specimen report back to you? How will you want to receive the results? Do you think family members should be informed of the result if it may impact on their health? Why do you think so? |
| 8. | **Perception of community participation in research** | What do you expect from researchers who want to conduct research in your community? Do you know of any customary norms they need to comply with? Will you want to have a say in which type of research is conducted in your community? Which part of the research will you want to have a say? How will you want researchers to go about involving you in their research? |

Thank you for choosing to participate and for your time.
